# The relationship between neurocognitive performance and HRV parameters in nurses and non‐healthcare participants

**DOI:** 10.1002/brb3.2481

**Published:** 2022-02-22

**Authors:** Taryn Chalmers, Shamona Eaves, Ty Lees, Chin‐Teng Lin, Phillip J. Newton, Roderick Clifton‐Bligh, Craig S. McLachlan, Sylvia M. Gustin, Sara Lal

**Affiliations:** ^1^ Neuroscience Research Unit University of Technology Sydney Sydney New South Wales Australia; ^2^ Neuroscience Research Unit School of Life Sciences University of Technology Sydney Sydney New South Wales Australia; ^3^ The Pennsylvania State University Department of Human Development and Family Studies University Park Pennsylvania USA; ^4^ Computational Intelligence and Brain Computer Interface Centre (CIBCI) FEIT University of Technology Sydney Sydney New South Wales Australia; ^5^ School of Nursing & Midwifery Western Sydney University Penrith South New South Wales Australia; ^6^ Medicine Northern Clinical School Kolling Institute of Medical Research St Leonards New South Wales Australia; ^7^ Centre for Healthy Futures Torrens University Australia ‐ Sydney Campus Pyrmont Pyrmont New South Wales Australia; ^8^ University of New South Wales School of Psychology Sydney New South Wales Australia; ^9^ Faculty of Science University of Technology Sydney Sydney New South Wales Australia

**Keywords:** cognitive performance, electrocardiography, heart rate variability, mental health, nurses

## Abstract

Nurses represent the largest sector of the healthcare workforce, and it is established that they are faced with ongoing physical and mental demands that leave many continuously stressed. In turn, this chronic stress may affect cardiac autonomic activity, which can be non‐invasively evaluated using heart rate variability (HRV). The association between neurocognitive parameters during acute stress situations and HRV has not been previously explored in nurses compared to non‐nurses and such, our study aimed to assess these differences. Neurocognitive data were obtained using the Mini‐Mental State Examination and Cognistat psychometric questionnaires. ECG‐derived HRV parameters were acquired during the Trier Social Stress Test. Between‐group differences were found in domain‐specific cognitive performance for the similarities (*p* = .03), and judgment (*p* = .002) domains and in the following HRV parameters: SDNN_baseline,_ (*p* = .004), LF_preparation_ (*p* = .002), SDNN_preparation_ (*p* = .002), HF_preparation_ (*p* = .02), and TP_preparation_ (*p* = .003). Negative correlations were found between HF power and domain‐specific cognitive performance in nurses. In contrast, both negative and positive correlations were found between HRV and domain‐specific cognitive performance in the non‐nurse group. The current findings highlight the prospective use of autonomic HRV markers in relation to cognitive performance while building a relationship between autonomic dysfunction and cognition.

## Graphical abstract

The association between neurocognitive parameters during acute stress situations and HRV has not been previously explored in nurses compared to non‐nurses and such, our study aimed to assess these differences. The current findings highlight the prospective use of autonomic HRV markers in relation to cognitive performance while building a relationship between autonomic dysfunction and cognition.

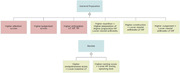

## 1 INTRODUCTION

Nurses represent the largest sector of the healthcare workforce (Australian Institute of Health & Welfare, 2016), spending more time with patients than any other primary care professional (Schroeder & Lorenz, 2018). They regularly experience a variety of work‐related stressors including, but not limited to, high workloads, time constraints, long work hours, irregular schedules, and a lack of professional support/resources (Gong et al., 2014; Letvak et al., 2012; Lim et al., 2010; Maharaj et al., 2019; Pikó, 1999). These work stressors see nurses regularly experiencing both acute and chronic stress and as a result, the cardiac sympathetic nervous system may be hyperactivated (Kim et al., 2018). Finally, given that mental fatigue and workplace stressors have been implicated in reduced healthcare outcomes (Lim et al., 2010), identifying areas of cognitive fatigue and its impact on physiological outcomes is of practical importance to the community. Given the reported high levels of acute and chronic stress within the nursing occupation, this cohort was selected to represent a stress population, in comparison to the general population, which is made up of non‐healthcare professionals.

Heart rate variability (HRV) can be used to assess cardiac autonomic responses to physiological and environmental changes. These environmental changes are then followed by an adaptive cardiac autonomic response (Kim et al., 2018). Reductions in total HRV have been associated with impaired regulatory and homeostatic autonomic functions, representing a reduction in the body's ability to cope with internal and external stressors (Kim et al., 2018). In most studies, HRV variables change in response to stress—with the most frequently reported change being reduced parasympathetic activity, which is characterized by a decrease in the HF activity and an increase in the LF activity. Furthermore, reduced HRV has been increasingly suggested to be associated with risk factors for cognitive impairment such as high cholesterol (Christensen et al., 1999; Felber Dietrich et al., 2006), hypertension (Schroeder et al., 2003), diabetes mellitus (Carnethon et al., 2003), and depression (Yeragani et al., 1991). Further, reduced HRV has been increasingly associated with increased levels of inflammation and reduced telomere length (Ask et al., 2018; Williams et al., 2019), which have been increasingly associated with cognitive decline and dementia (Devore et al., 2011; King et al., 2018). Yet, more direct associations between HRV and cognitive functioning remain less well established (Zeki Al Hazzouri et al., 2014).

The interactions of cognition, stress, and cardiac autonomic responses are of interest. Hansen et al. (2003) found that higher levels of HRV associated with stress permitted better working memory and executive functioning. Similarly, Colzato et al. (2018) and Hovland et al. (2012) found that higher resting HRV was associated with better cognitive flexibility and executive function. Additionally, low HRV has been linked to poorer attention and attentional control (Johnsen et al., 2003; Park et al., 2012). Frewen et al. (2013) assessed HRV and cognition in a large longitudinal study, noting that reduced HRV was associated with poorer global cognitive performance in older adults, which does limit its extrapolation to younger populations. In contrast, lower HRV has also been associated with better reaction time (Hansen et al., 2004; Wang et al., 2005). Additionally, other research has associated higher HRV to higher prefrontal cortex (PFC) functioning (Luft et al., 2009; Maier & Hare, 2017), which may further implicate executive functions and selective attention (Thayer & Brosschot, 2005; Thayer & Lane, 2009) as the PFC is well known to be significantly involved in various cognitive functions such as decision making, memory, conflict monitoring and error detection (Botvinick et al., 2004; Posner et al., 2007).

Nurses, in particular, are required to complete tasks efficiently and without error; making it important to explore variations in, and relationships between, physiological and cognitive parameters. However, despite the potential associations between HRV and cognitive performance, this area remains poorly understood and rarely explored, especially in healthcare populations. Therefore, the aim of this study was to analyze the relationship between autonomic regulation, as measured by heart rate variability, and cognitive processes in nurses and nonhealthcare working groups. It is hypothesized that the nursing cohort will 1) have significantly higher resting LF HRV when compared to the general population; 2) will show significant associations between LH HRV and the speaking task; and 3) will show significant relationships between HF HRV and the resting phase.

## 2 MATERIALS AND METHODS

### 2.1 Participants and sampling

A cross‐sectional study design was used to examine the relationship between HRV and cognition in a cohort of 30 clinically active Australian nurses and 50 nonhealthcare (NHC) participants aged between 18–45 years (mean age 29.2 ± 6.0 and 25.3 ± 4.0, 90% female and 56% female, respectively). In order to be classified as an NHC worker, and thus included in the general population, the NHC group must not be employed in any healthcare‐related occupation. Participants were recruited from the general public with advertisements and posters placed around the university and social media advertisements undertaken in online nursing forums. Prior to inclusion, an in‐house designed lifestyle questionnaire adapted from the Lifestyle Appraisal Questionnaire (LAQ; Craig et al., 1996) was utilized to screen participants for medication use, alcohol intake (over 16 standard drinks a day), smoking habits (over 10 cigarettes a day), and chronic disease/illness. Participants were excluded if they answered affirmatively to any of the screening questions mentioned above. Participants were asked to refrain from consuming coffee and alcohol for 24 hr prior, and from smoking cigarettes for 12 hr prior to the study. Further, participants were required to have had a minimum of 8 hr of sleep in the evening preceding the study. The study had institutional ethics approval from the University of Technology Sydney Human Research Ethics Committee, and all participants provided signed informed consent prior to the commencement of the study.

### 2.2 Experimental procedures

At the commencement and conclusion of each session, blood pressure (BP) measurements were taken three times with an automated blood pressure monitor (Omron IA1B, Japan), and averaged to confirm inclusion into the study (BP < 160/100 mm Hg). Demographic and lifestyle data were collected using an in‐house questionnaire adapted from the LAQ (Craig et al., 1996). Additionally, work‐related data such as time in role, shiftwork, and job satisfaction was also collected using an in‐house designed questionnaire. A modified Trier Social Stress Test (TSST; Kirschbaum et al., 1993) was then utilized to elicit a controlled stress response in participants. For this section of the study, participants underwent a 10 min resting baseline session, followed by the TSST that constituted of a 10 min preparation/anticipation task where participants were required to prepare a short speech, followed by a 5 min public speaking task, and finally a 5 min mental arithmetic task. During baseline and each component of the TSST, 3‐lead ECG data were captured using disposable Ag/AgCl electrodes placed on the participant's upper torso under each clavicle on the coracoid processes, and one just below the sternum over the xiphoid process (Combatalade, 2013). The ECG was recorded during the 10‐min resting state (subject sitting quietly with eyes open), and then again during each of the three stress tasks; 10‐min anticipation task, 5‐min public speaking task, and 5‐min mental arithmetic task. The study was undertaken in a controlled laboratory environment, with temperature, light sources, and external sound all controlled for during the study.

Following the TSST, the Mini Mental State Examination (MMSE; Folstein et al., 1983) and Cognistat (Mueller et al., 2007) were administered to provide measures of both global and domain‐specific cognitive performance.

### 2.3 ECG data processing

Raw ECG data were processed utilizing the Kubios HRV Premium software (Version 3.1.0, Kubios Oy, Finland) to generate HRV parameters. Frequency domain activity was calculated Welch's periodogram method (Welch, 1967) for the following HRV frequency bands: low frequency (LF) power (0.04–0.15 Hz), high frequency (HF) power (0.15–0.4 Hz), and total power (TP), as well as the LF:HF ratio. Additionally, time domain activity was calculated for the standard deviation of NN intervals (SDNN). It should be noted that in the process of deriving these variables, both visual and automatic artifact correction process was undertaken (Tarvainen et al., 2014), as well as the Smoothn Priors method (Tarvainen et al., 2002) of trend component rejection were utilized, and HRV data were log‐transformed prior to analysis where relevant.

### 2.4 Statistical analysis

Statistical analysis was performed using SPSS Version 23.0 (SPSS Inc.; Chicago, IL, USA) with statistical significance defined as *p* < .05. Data were initially subject to descriptive statistics. Independent sample *t*‐tests were then used to identify differences in demographic and psychometric data, HRV measures, and performance on the cognitive tasks between the two groups. As multiple comparisons were made, a Holm–Bonferroni correction was then applied to avoid type I errors with the family‐wise α (a) level set at .05. Following that, partial Pearson's correlation analysis (controlling for age, body mass index (BMI), and total years of education) was utilized to determine associations between HRV parameters and demographics, psychometrics, and cognitive measures during the baseline, preparation, and two stress tasks of the TSST. Where three or more significant relationships to a dependent variable were found, a multiple linear regression analysis was utilized to further examine the relationship between HRV and dependent variables.

Additionally, the least absolute shrinkage and selection operator (LASSO) analysis (Tibshirani, 1996) was also used to provide a further investigation of the relationship between HRV and cognitive performance and inform the multiple regression analysis. LASSO analysis was computed using Matlab (Version 2018b, Mathworks, USA), as per the following equation:

$$\begin{array}{c} min\\ \beta_{0}\beta \end{array}\left(\frac{1}{2N}\sum_{i\;=\;1}^{N}{\left(y_{i}-\;\beta_{0}-x_{i}^{T}\beta \right)}^{2}+\lambda \sum_{j\;=\;1}^{p}\left|\beta_{j}\right|\right)$$

where:

‐ *N* is the number of observations.
‐ *y_i_* is the response at observation *i*.
‐ *x_i_* is data, a vector of *p* values at observation *i*.
‐ *λ* is a nonnegative regularization parameter corresponding to one value of Lambda.
‐ The parameters *β_0_* and *β* are scalar and *p*‐vector, respectively.
‐ As *λ* increases, the number of nonzero components of *β* decreases.
‐ The lasso problem involves the *L* ^1^ norm of *β*, as contrasted with the elastic net algorithm.

## 3 RESULTS

### 3.1 Demographics

Data from 30 clinically active nurses and 50 nonhealthcare (NHC) participants were included in the current study. Average data and *p*‐values for group demographics and psychometrics are presented in Table 1. All significant findings remained significant following post hoc corrections. Significant between‐group differences were seen for age (nurses: 29.2 ± 6.0 years, NHC group: 25.3 ± 4.0 years, *p* = .001), but not BMI, years of education, or blood pressure. Blood pressure measurements were also within a normal range for both groups.

**TABLE 1:** Comparison of demographic and psychometric data (n = 80)

	Nurses n = 30	Nonhealthcare n = 50	
	Mean ± SD or %	Mean ± SD or %	*p*‐Value
Age	29.2 ± 6.0	25.3 ± 4.0	.001^*^
Sex (Female)	90% (27F)	56% (28F)	n/a
Body Mass Index	25.1 ± 3.8	24.0 ± 3.2	.14
Total Years of Education	16.6 ± 1.4	15.8 ± 2.1	.07
Pre Systolic BP	114.5 ± 9.7	113.3 ± 10.1	.61
Pre Diastolic BP	73.5 ± 9.2	74.0 ± 8.4	.84
Pre Heart Rate	66.4 ± 8.9	69.5 ± 9.3	.17
Post Systolic BP	114.6 ± 8.0	115.0 ± 8.7	.83
Post Diastolic BP	72.0 ± 8.1	71.3 ± 8.4	.70
Post Heart Rate	66.5 ± 6.0	67.2 ± 8.2	.66
Position: RN/Midwife	93.4% (28)	n/a	n/a
Position: Other	6.6% (2)	n/a	n/a
Facility: Hospital	96.7% (29)	n/a	n/a
Facility: Other	3.3% (1)	n/a	n/a
Shift workers	96.7% (29)	n/a	n/a
Job satisfaction: Yes	70% (21)	n/a	n/a
Job satisfaction: No	30% (9)	n/a	n/a
Depression Score	5.7 ± 6.4	3.9 ± 4.8	.17
Anxiety Score	6.4 ± 6.5	3.2 ± 4.6	.012^*^

Abrbeviations: BP, blood pressure in bpm (beats per minute); RN, registered nurse; SD, standard deviation. *Note*: *p*‐Values marked with an asterisk remained significant following post hoc correction.

With regards to work characteristics (Table 1), the Nurse cohort composed of predominantly females who were employed as Registered Nurses (RN's) and who worked in hospital settings, which is well reflective of the current nursing population in Australia and is also dominantly females working as registered nurses in hospital settings (Health, 2017). The majority of the nurses (96.7%) also engaged in shift work and reported positive job satisfaction.

### 3.2 Differences in HRV and cognitive measures between groups

Between‐group differences in cognitive performance and HRV measures (means ± SD and *p*‐values) are presented in Table 2. Values marked with an asterisk indicate findings that remained significant following post hoc correction. Significant differences were found for similarities (*p* = .03), and judgment (*p* = .002)—of which the nurse group performed slightly poorer in these cognitive tasks. Additionally, the nurse cohort fell below the impairment threshold for the judgment domain (averaging below 4 points). Performance in each of the other cognitive domains was in the normal range for both groups.

**TABLE 2:** Comparison of cognitive and HRV variables between groups (n = 80)

Variable (impairment threshold)/HRV parameter	Nurses (n = 30)	Nonhealthcare (n = 50)	
	Mean ± SD	Mean ± SD	*p*‐Value
MMSE total (< 25)	29.3 ± 1.0	29.5 ± 0.8	.22
Cognistat: Attention (< 6)	7.5 ± 0.9	7.8 ± 0.5	.047
Cognistat: Comprehension (< 5)	5.9 ± 0.4	5.8 ± 0.4	.62
Cognistat: Repetition (< 11)	11.7 ± 0.8	11.9 ± 0.5	.19
Cognistat: Naming (< 7)	7.7 ± 0.6	7.9 ± 0.4	.13
Cognistat: Construction (< 4)	5.7 ± 0.8	5.9 ± 0.6	.13
Cognistat: Memory (< 10)	10.9 ± 1.6	11.5 ± 1.3	.12
Cognistat: Calculation (< 3)	4.0 ± 0	4.0 ± 0	.20
Cognistat: Similarities (< 5)	7.2 ± 1.2	7.7 ± 0.6	.03^*^
Cognistat: Judgment (< 4)	3.4 ± 2.0	4.8 ± 1.7	.002^*^
Baseline LF	6.7 ± 0.7	7.0 ± 0.8	.04
Baseline HF	5.8 ± 0.8	6.0 ± 1.0	.30
Baseline LF:HF	2.82 ± 1.4	3.2 ± 2.1	.37
Baseline TP	17.1 ± 2.0	18.1 ± 2.5	.07
Baseline SDNN	39.8 ± 12.1	46.4 ± 14.9	.004^*^
Preparation task LF	6.7 ± 0.6	7.2 ± 0.7	.002^*^
Preparation task HF	5.8 ± 0.7	6.2 ± 0.9	.02^*^
Preparation task LF:HF	2.9 ± 1.7	3.1 ± 1.8	.69
Preparation task TP	17.1 ± 1.7	18.6 ± 2.1	.003^*^
Preparation task SDNN	38.3 ± 10.2	48.9 ± 15.8	.002^*^
Speaking task LF	7.0 ± 0.7	7.1 ± 0.7	.37
Speaking task HF	5.9 ± 0.9	6.1 ± 0.9	.29
Speaking task LF:HF	4.0 ± 3.9	3.3 ± 2.2	.31
Speaking task TP	17.5 ± 2.0	18.0 ± 2.0	.32
Speaking task SDNN	43.9 ± 13.7	46.8 ± 13.4	.36
Mental arithmetic LF	7.0 ± 0.6	7.1 ± 0.6	.36
Mental arithmetic HF	5.9 ± 0.9	6.1 ± 0.9	.15
Mental arithmetic LF:HF	3.8 ± 2.6	3.5 ± 3.3	.64
Mental arithmetic TP	17.7 ± 1.8	18.0 ± 1.8	.38
Mental arithmetic SDNN	43.6 ± 14.0	47.8 ± 12.8	.18

Abbreviations: HF, high frequency; HRV, heart rate variability; LF, low frequency; LF:HF, ratio between low frequency power and high frequency power (%); MMSE, Mini Mental State Examination; SD, standard deviation; SDNN, standard deviation of NN intervals (ms^2^); TP, total power.

*Note*: *p*‐Values marked with an asterisk remained significant following post hoc correction.

Significant differences were found in the following HRV parameters between the two groups: SDNN_(Baseline, Preparation)_ (*p* = .004, *p* = .002), LF_(Preparation)_ (*p* = .002), HF_(Preparation)_ (*p* = .02), and TP_(Preparation)_ (*p* = .003)—with the nursing group showing lower HRV measures in all instances.

#### 3.2.1 Associations between HRV and cognitive performance/psychometrics

Negative correlations were found between HF_(Baseline)_ and comprehension performance (*r* = ‐.41, *p* = .03) and HF_(Speaking)_ and naming performance (*r* = −.39, *p* = .04).

Significant correlations between HRV parameters and cognition for the NHC group are displayed in Table 4. Significant negative correlations were found between cognitive domains and multiple LF, HF, and TP parameters. Conversely, positive correlations were found between the ESS and LF:HF ratio during the preparation (*r* = .36, *p* = .01) and speaking task (*r* = .39, *p* = .007). Positive correlations were also found for LF_(Preparation)_ and repetition performance (*r* = .29, *p* = .045), HF during the preparation task and repetition performance (*r* = .30, *p* = .04), and HF during mental arithmetic and preparation performance (*r* = .33, *p* = .02). However, an inverse relationship was found between the LF:HF_(Arithmetic)_ ratio and performance in the repetition (*r* = −.51, *p* ≤ .001), construction (*r* = −.49, *p* ≤ .001), and judgment (*r* = −.49, *p* = .001) domains.

#### 3.2.2 LASSO analysis between HRV and cognitive performance/psychometrics

The relationship between HRV and cognitive performance in each group was further explored using LASSO analysis. In the nursing group, the LASSO analysis indicated the importance of the relationship between TP_(Baseline)_ and the naming, construction, and judgment domains when utilizing a cut‐off normalized weight of an absolute value of 0.75. Links were also found between HF_(Preparation, Speaking)_, and naming performance, TP_(Speaking)_ and naming performance, LF:HF_(Speaking)_ and naming performance, and LF:HF_(Arithmetic)_ and stress levels (as measured by the DASS). In the nonhealthcare group, the LASSO analysis indicated the importance of the relationship between LF:HF_(Speaking)_ and Memory performance, LF power_(Arithmetic)_ and Judgment performance, and LF:HF_(Arithmetic)_ and Judgment performance when utilizing a cut‐off normalized weight of an absolute value of 0.75.

### 3.3 Multiple regression analysis

As multiple dependent variables were associated with LF:HF_(Arithmetic)_ in the NHC group as informed by the correlation analysis (seen in Table 3), multiple regression analysis was performed to determine predictive capability. Additionally, multiple dependent variables were associated with TP_(Baseline)_ in the nursing group as informed by the LASSO analysis and hence was also followed by multiple regression to identify the strongest predictors of TP in the nursing group.

**TABLE 3:** Associations between HRV parameters and psychometrics/cognitive performance in nurses (n = 30)

HRV parameter	Measure/cognitive domain	*r*	*p*‐Value
Baseline HF	Comprehension	‐.41	.03*
Speaking Task HF	Naming	‐.39	.04*

Abbreviation: HF, high frequency.

* Statistical significance: *p* < .05.

**TABLE 4:** Associations between HRV parameters and psychometrics/cognitive performance in NHC professionals (n = 50)

HRV parameter	Measure/cognitive domain	*R*	*p*‐Value
Preparation Task LF	Repetition	.29	.045*
Preparation Task HF	Repetition	.30	.04*
Mental Arithmetic LF:HF	Repetition	‐.51	<.001*
	Construction	‐.49	<.001*
	Judgment	‐.49	.001*

Abbreviations: HF, high frequency; LF, low frequency; LF:HF, low frequency to high frequency ratio (%).

* Statistical significance: *p* < .05.

The regression analysis for LF:HF_(Arithmetic)_ in the NHC (Table 5) was significant (*p* < .001) retaining three of the four originally entered variables (repetition (*p* < .001), construction (*p* = .001), and judgment (*p* = .03)). The regression model explained 58.3% of the variance in LF:HF_(Arithmetic)_ in the NHC group (F = 15.7, *p* < 0.001; *R* = 0.76, *R* ^2^ = 0.58; adjusted*R* ^2^ = 0.55).

**TABLE 5:** Multiple regression analysis for LF:HF_(Arithmetic)_ in the nonhealthcare group (n = 50)

Regression analysis for LF:HF_(Arithmetic)_ *R* = 0.76, *R* ^2^ = 0.58, adjusted *R* ^2^ = 0.55, F = 15.7, *p* < .001*					
Variable	B	SE	β	t	*p*‐Value
Repetition	−3.53	.73	−.48	−4.87	<.001
Construction	−2.24	.60	−.39	−3.72	.001
Judgment	−.476	.21	−.25	−2.30	.03

Abbreviations: β, beta; LF:HF, low frequency to high frequency ratio (%); SE, standard error.

* Statistical significance: *p* < .05.

The regression analysis for TP_(Baseline)_ in the nursing group was overall non‐significant and did not retain any of the three originally entered variables.

## 4 DISCUSSION

In this exploratory study, significant between‐group differences were noted in the cognitive domains of similarities, and judgment, in which the nurses performed poorer than controls. Nurse performance in the judgment domain also fell below the expected range into mild impairment. Poorer judgment (which is a sub‐domain of reasoning) in the nursing group raises concerns as in clinical settings it may be an indicator for both poor reasoning and decision making which may affect clinical output and patient outcomes (Benner et al., 2008).

Additionally, significant between‐group differences were noted in LF, HF, TP, and SDNN power where the nurses portrayed consistently lower HRV than the NHC's. Lower LF power is suggestive of a lower baroreflex response to the TSST (Goldstein et al., 2011). The arterial baroreflex response buffers acute fluctuations in blood pressure that occur during postural changes and stress. An impaired baroreflex response may subsequently be indicative of long‐term blood pressure dysregulation (Heusser et al., 2005). Lower HF and TP are suggestive of less parasympathetic activity/parasympathetic withdrawal and lower overall autonomic input that is an integral component of autonomic imbalance (Olshansky et al., 2008). Reduced SDNN also indicates autonomic dysfunction as reductions may reflect abnormalities in sympathetic and parasympathetic activity (Nolan et al., 1998). Fluctuations in HRV may also be indicative of individuals who are able to better regulate their autonomic functions. Individuals who have a higher HRV activity may have greater cardiovascular fitness and be more resilient to stress, whilst those with lower HRV have poorer cardiovascular fitness and are less resilient to stress (Campos, 2017). As the nursing cohort showed consistently lower HRV compared to controls, it may reflect poorer cardiovascular fitness and stress resilience.

A number of previous studies suggest that autonomic cardiovascular factors (such as HRV) may be associated with cognition (Frewen et al., 2013; Zeki Al Hazzouri et al., 2014). However, this body of literature is limited and mostly confined to assessing attention and executive functioning. The present study found that HF was inversely related to comprehension and naming performance in nurses; with higher HF reflecting parasympathetic dominance. This association aligns with the early results of Hansen et al. (2003) who reported a connection between resting HF‐HRV, processing speed, and the accuracy of responses to monitoring tasks. The study found participants with high HF‐HRV performed better than participants with low HF‐HRV. These results were also confirmed during conditions of a threat of shock (Hansen et al., 2009).

Further, comprehension and naming both require the understanding/expression of information, and the ability to easily access and manipulate stored knowledge (Wagner et al., 1994). Increased parasympathetic activity has been associated with improved cognitive functioning and capacity, possibly due to a larger availability of cognitive resources (Bruya & Tang, 2018; Thayer & Lane, 2009). It should be noted, however, that although more cognitive resources may be available during parasympathetic dominance, studies have suggested the neuronal allocation of these resources may be primarily directed towards basic biological functions such as digestion, baseline threat awareness, and circadian rhythms (Melis & van Boxtel, 2007).

In contrast, in the non‐healthcare group, positive relationships were found between both LF and HF power and repetition performance. Increased HRV has previously been associated with improved memory, attention, and cognitive flexibility (Colzato et al., 2018; Hansen et al., 2004; Hansen et al., 2003; Hovland et al., 2012). While higher LF activity predominantly reflects sympathetic dominance, small amounts of sympathetic modulation seen with minor levels of stress may be beneficial to cognition (Duncko et al., 2007), consistent with an inverted U‐shaped hypothesis. Flexible PNS adaptations (both withdrawal and enhancement) have also been associated with cognitive and neural improvements (Lin et al., 2017). Increased parasympathetic (HF) activity and improved repetition performance are consistent with the neurovisceral integration model. The model suggests that greater parasympathetic activity is a marker of increased cognitive capacity and functioning due to the high degree of interconnectedness between neural and peripheral structures that regulate behavior and physiological states (Giuliano et al., 2018; Thayer & Lane, 2009; 2000). Similar to the SNS, higher PNS activity may also lead to improvements in cognitive processes via the release of cholinergic neurotransmitters (Clark et al., 1999) and serotonin (Meneses & Liy‐Salmeron, 2012) that play a role in memory, sentence recognition, and learning (Meneses, 2015). Thus, increased LF and HF may be associated with better repetition performance in non‐health professionals due to the close relationship between eustress and distress and neurohormone release associated with the SNS and PNS.

LF:HF ratio increased as repetition, construction, and judgment decreased in the nonhealthcare group. Elevated LF:HF ratio is indicative of sympathovagal cardiac autonomics associated with the withdrawal of vagal tone and increased sympathetic activity. Though not commonly investigated, sympathetic exacerbation has been linked with Alzheimer's (de Vilhena Toledo & Junqueira Jr, 2008) and increases in sympathetic tone have been associated with cognitive impairment in individuals with diabetes and hypertension (Auroprajna et al., 2018). As we have shown, heightened sympathovagal balance was associated with poorer domain‐specific cognitive performance in relation to language skills (repetition), visuospatial ability (construction), and judgment. It could be suggested that acute stress is associated with cognitive decline that is also associated with increased cardiac sympathetic activity.

### 4.1 Limitations and future directions

Due to the cross‐sectional nature of the study, only short‐term variations in HRV and cognitive performance could be assessed. Further research implementing longer‐term measurements in HRV and cognition could potentially track ongoing changes in cognitive performance or a point of cognitive decline. Technological advances may allow for HRV assessments via wearable technologies (Reali et al., 2019) that would allow for easier wear in everyday circumstances while continuously assessing cardiac autonomic fluctuations. It could also account for both acute and chronic stress and even fatigue. If HRV parameters are able to predict declines in cognition, this could also lead to the non‐invasive monitoring of cognitive performance in the workplace, which could in turn reduce complications or errors associated with cognitive decline. Further, given the identification of a number of trends in the data, increasing the sample size would allow for more robust statistical analysis and strengthening of the statistical findings. There was also a substantial gender difference in the two cohorts (the nursing cohort had a higher number of females than the NHC group). Although research suggests that age has a significantly greater influence on HRV parameters than gender, future research may benefit from ensuring gender‐matched cohorts for comparison. Finally, it would be prudent in future studies, to stratify the general population group based on occupation to reduce the heterogeneity of the group and allow for stronger comparisons based on occupation and perceived occupational stress.

## 5 CONCLUSIONS

It is well‐established that nurses experience ongoing physical and mental strain, leaving many stressed and potentially leading to autonomic dysregulation (Kim et al., 2018; Maharaj et al., 2019). The present study found between‐group differences in domain‐specific cognitive performance and HRV between nurses and NHCs. Furthermore, the dynamic relationship between HRV and cognition/psychometrics was also observed in nurses and NHCs who demonstrated several relationships to domain‐specific cognitive performance. The present findings contribute to and strengthen the understanding of the potential relationships between autonomic dysfunction and cognitive function, and HRV and stress‐related dysfunction. Present results also highlight the prospective use of autonomic markers in relation to cognitive performance and psychometrics. However, given the exploratory nature of the study, replication with a larger sample size should be undertaken to ensure statistical robustness of the identified relationships.

Continuing research in this area will not only ensure the health and well‐being of nursing and other healthcare professionals but could also lead to possible predictors of cognitive impairment, enabling better targeted preventive strategies and public health measures. Such research may allow us to detect acute stress responses that may interfere with cognition—thus we may be able to detect and avoid inducing errors, which in occupations such as nursing, outcomes are high risk. Importantly, such research may be also utilized in other high‐stress populations, such as doctors, first responders (e.g. police, paramedics, and firefighters), and defense personnel who due to their inherently stressful occupations, may also be at increased risk of autonomic dysfunction and/or cognitive impairment.

## CONFLICTS OF INTEREST

All authors disclose no actual or potential conflicts of interest including any financial, personal, or other relationships with other people or organizations that could inappropriately influence (bias) their work. None of the data or materials for the experiments reported here is available, and none of the experiments were preregistered.

### PEER REVIEW

The peer review history for this article is available at https://publons.com/publon/10.1002/brb3.2481

## Data Availability

None of the data or materials for the experiments reported here is available, and none of the experiments were preregistered.
